# Early prediction matters: renal biomarkers in coronary artery bypass grafting

**DOI:** 10.1590/2175-8239-JBN-2025-E011en

**Published:** 2025-07-18

**Authors:** Renata de Souza Mendes

**Affiliations:** 1 Universidade do Estado do Rio de Janeiro, Rio de Janeiro, RJ, Brazil.; 2 Universidade Federal do Rio de Janeiro, Rio de Janeiro, RJ, Brazil.

## Acute Kidney Injury and Cardiac Surgery

Acute kidney injury (AKI) remains one of the most common and severe complications following cardiac surgery, with an incidence ranging from 5 to 43% depending on the population and diagnostic criteria used^1,2^. Importantly, the development of AKI is independently associated with a 3- to 8-fold increase in perioperative mortality^3^, as well as prolonged intensive care and hospital stays, with consequential increases in healthcare costs^3^.

**Figure 1:**
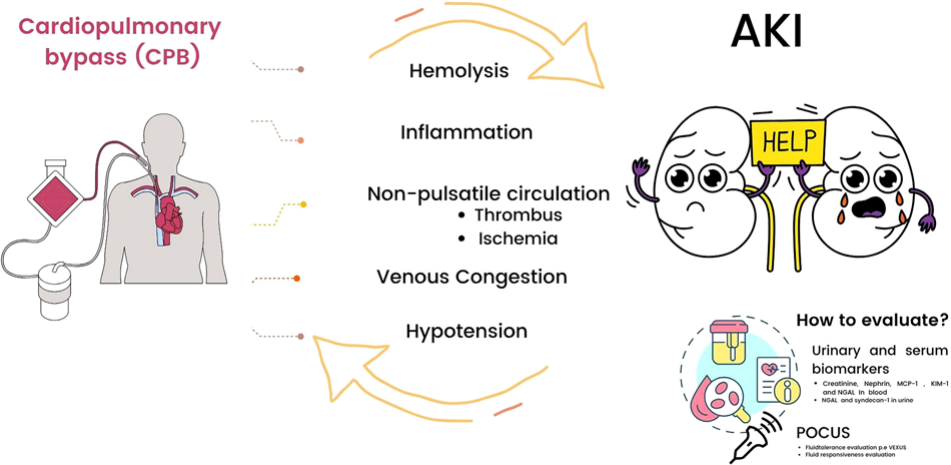
Pathophysiological Mechanisms of Cardiopulmonary Bypass Contributing to Acute Kidney Injury.

The pathogenesis of cardiac surgery-associated AKI is complex and multifactorial, encompassing hemodynamic instability, systemic inflam­mation, ischemia-reperfusion injury, cardiopulmonary bypass (CPB)-related factors such as ischemia, hemolysis, oxidative stress, non-pulsatile flow, and hemodilution, as well as venous con­gestion and nephrotoxic drug exposure^4^. A critical mechanism underlying AKI in this setting is renal hypoperfusion, particularly affecting the renal medulla, which is highly susceptible to ischemic injury due to its inherently low oxygen supply and limited vascularization^2^. Figure 1.

Abbreviations – CPB, Cardiopulmonary bypass; AKI, acute kidney injury; NGAL, Neutrophil Gelatinase-Associated Lipocalin; KIM-1, Kidney Injury Molecule-1; MCP-1, Monocyte Chemoattractant Protein-1; VEXUS, Venous Excess Ultrasound Score; POCUS, Point of Care Ultrasound.

## Cardiopulmonary Bypass and Renal Vulnerability

CPB further increases AKI risk by promoting non-physiological perfusion patterns, including suboptimal flow and pressure, non-pulsatile circulation, and potential for thrombus formation and rewarming-related injury^5,6^. Additionally, low cardiac output and hypotension contribute to impaired renal perfusion, further exacerbated by the activation of vasoconstrictive pathways, thereby compounding the hemodynamic insult^7^.

## Biomarkers in AKI: Limitations and Future Directions

The diagnosis of AKI still depends on delayed markers, like serum creatinine and urine output, indicating established damage, similar to the diagnosis of myocardial infarction after necrosis. In contrast to early detection by troponins in cardiac ischemia, there is no analogous early warning system for kidney damage, limiting timely intervention. In addition, the KDIGO criteria for AKI diagnosis may be unreliable after cardiac surgery because of the influence of fluid resuscitation on diagnostic markers^8^.

This reality underlines the urgent need for sensitive and specific biomarkers that can detect subclinical renal injury, ideally before irreversible damage occurs. The emergence of urinary biomarkers that reflect glomerular and tubular stress—such as nephrin, NGAL, KIM-1, and MCP-1—is particularly promising in surgical scenarios where the timing of renal insult is clearly defined, such as during CPB.

In this issue of the Brazilian Journal of Nephrology^9^, the study entitled “Urinary and serum biomarkers of renal injury in coronary artery bypass grafting: a prospective evaluation with new biomarkers” investigates the impact of CPB on the release of urinary and systemic biomarkers of kidney and endothelial injury. This prospective single-center study enrolled patients undergoing coronary artery bypass grafting (CABG), comparing those who underwent the procedure with those without CPB.

Over a 15-month period, the investigators evaluated 22 patients over 18 years of age, excluding those with pre-existing chronic kidney disease, valvular disease, prior cardiac surgery or transplant, or recent exposure to nephrotoxic agents. Biomarker quantification was performed using ELISA techniques. The authors measured urinary nephrin, KIM-1, MCP-1, and NGAL and serum syndecan-1 (a marker of endothelial glycocalyx degradation) and NGAL.

Of the total cohort, 9 patients (41%) underwent CPB. Although the sample size is limited and only univariate analyses were performed, the study provides critical insight: baseline biomarker levels (before surgery) were similar between groups, but following the surgical procedure, there was a significant increase in both urinary nephrin and NGAL (urinary and serum) in patients undergoing CPB. These findings support the hypothesis that CPB may play a direct role in triggering renal and endothelial injury, contributing to the multifactorial nature of AKI in this context.

However, some methodological limitations should be mentioned. The CPB group had higher STS (The Society of Thoracic Surgeons Operative Risk Calculator) scores, more pronounced left ventricular dysfunction, and likely consisted of patients with more complex coronary anatomy (e.g., multivessel disease, diffuse atherosclerosis, small-caliber or inaccessible arteries). These characteristics inherently increase procedural risk and may act as confounders. Moreover, it was not clearly explained how the decision to assign patients to CPB or off-pump CABG was made, raising the possibility of selection bias. Since the choice of the surgical strategy likely reflects the complexity of the case and the underlying cardiovascular status, this non-randomized allocation may have impacted the observed outcomes, particularly regarding biomarker elevation and AKI risk.

Additionally, the use of the CKD-EPI equation in the acute setting, while commonly applied, is not fully validated for the early detection of acute changes in renal function.

Despite these limitations, the study’s main strength lies in its ability to demonstrate differential biomarker release only after the initiation of CPB, reinforcing the potential role of CPB in contributing to kidney injury. The data presented offer a valuable contribution to the growing field of biomarker-based renal risk assessment in perioperative medicine.

## Toward Precision Medicine In AKI: Stratification and Prevention

It is important that nephrology moves toward precision medicine, particularly in clinical scenarios involving multiple concurrent injurious stimuli, such as cardiac surgery. Identifying the predominant insult in a given patient may enable targeted interventions, mitigate renal injury more effectively, and ultimately improve outcomes. In this context, biomarkers are not only tools for early detection, but also for pathophysiological discrimination, risk stratification, and personalized therapeutic planning.

Future studies with larger sample sizes and multivariate adjustment for confounding variables are essential. Moreover, the inclusion of outcomes such as long-term renal recovery or need for renal replacement therapy would strengthen the clinical applicability of such findings.

As nephrologists, we must advocate for multidisciplinary strategies that incorporate biomarker surveillance, hemodynamic optimization, and kidney-sparing protocols in high-risk surgical patients. Early prediction must become a cornerstone of AKI management—transforming our approach from reactive to preventive.

## Data Availability

No data were generated or analyzed for this editorial.
