# Supplementary material for: Early prediction matters: renal biomarkers in coronary artery bypass grafting
Source: J Bras Nefrol. 2025 Jul 18;47(3):e2025E011. doi: 10.1590/2175-8239-JBN-2025-E011en (PMC12279284; doi:10.1590/2175-8239-JBN-2025-E011en)
Supplement: Translation

# Translation: Predição precoce é fundamental: biomarcadores renais na cirurgia de revascularização do miocárdio

**DOI:** 10.1590/2175-8239-JBN-2025-E011pt

**Authors:** Renata de Souza Mendes

**Affiliations:** 1 Universidade do Estado do Rio de Janeiro, Rio de Janeiro, RJ, Brasil.; 2 Universidade Federal do Rio de Janeiro, Rio de Janeiro, RJ, Brasil.

## Injúria Renal Aguda E Cirurgia Cardíaca

A injúria renal aguda (IRA) continua sendo uma das complicações mais comuns e graves após a cirurgia cardíaca, com uma incidência que varia de 5 a 43%, dependendo da população e dos critérios diagnósticos utilizados^1,2^. É importante ressaltar que o desenvolvimento de IRA está independentemente associado a um aumento de 3 a 8 vezes na mortalidade perioperatória^3^, bem como a cuidados intensivos prolongados e internações hospitalares, com consequente aumento nos custos de assistência médica^3^.

A patogênese da IRA associada à cirurgia cardíaca é complexa e multifatorial, abrangendo instabilidade hemodinâmica, inflamação sistêmica, lesão de isquemia-reperfusão, fatores relacionados à circulação extracorpórea (CEC), como isquemia, hemólise, estresse oxidativo, fluxo não pulsátil e hemodiluição, além de congestão venosa e exposição a drogas nefrotóxicas^4^. Um mecanismo crítico subjacente à IRA nesse contexto é a hipoperfusão renal, afetando particularmente a medula renal, a qual é altamente suscetível à injúria isquêmica devido ao seu suprimento inerentemente baixo de oxigênio e vascularização limitada^2^. Figura 1

**Figura 1:** Mecanismos Fisiopatológicos da Circulação Extracorpórea que Contribuem para a Injúria Renal Aguda.

Abreviações – CEC, circulação extracorpórea; IRA, injúria renal aguda; NGAL, lipocalina associada à gelatinase neutrofílica; KIM-1, molécula de lesão renal-1; MCP-1, proteína quimioatraente de monócitos-1; VEXUS, escore de ultrassom de excesso venoso; POCUS, do inglês “Point of Care Ultrasound”.

## Circulação Extracorpórea e Vulnerabilidade Renal

A CEC aumenta ainda mais o risco de IRA ao promover padrões de perfusão não fisiológicos, incluindo fluxo e pressão subótimos, circulação não pulsátil e potencial para formação de trombos e lesões relacionadas ao reaquecimento^5,6^. Além disso, o baixo débito cardíaco e a hipotensão contribuem para a perfusão renal inadequada, agravada pela ativação de vias vasoconstritoras, intensificando assim o impacto hemodinâmico^7^.

## Biomarcadores na IRA: Limitações e Direções Futuras

O diagnóstico de IRA ainda depende de marcadores tardios, como creatinina sérica e débito urinário, indicando dano estabelecido, semelhante ao diagnóstico de infarto do miocárdio após a ocorrência de necrose. Em contraste com a detecção precoce por troponinas na isquemia cardíaca, não existe um sistema de alerta precoce análogo para lesão renal, o que limita a intervenção oportuna. Além disso, os critérios do KDIGO para o diagnóstico de IRA podem ser pouco confiáveis após a cirurgia cardíaca, devido à influência da ressuscitação volêmica nos marcadores diagnósticos^8^.

Essa realidade ressalta a necessidade urgente de biomarcadores sensíveis e específicos que possam detectar a injúria renal subclínica, idealmente antes da ocorrência de danos irreversíveis. O surgimento de biomarcadores urinários que refletem o estresse glomerular e tubular – como nefrina, NGAL, KIM-1 e MCP-1 – é particularmente promissor em cenários cirúrgicos nos quais o momento do insulto renal é claramente definido, como durante a CEC.

Nesta edição do Jornal Brasileiro de Nefrologia^9^, o estudo intitulado “Biomarcadores urinários e séricos de injúria renal na cirurgia de revascularização do miocárdio: uma avaliação prospectiva com novos biomarcadores” investiga o impacto da CEC na liberação de biomarcadores urinários e sistêmicos de injúria renal e endotelial. Este estudo prospectivo de centro único incluiu pacientes submetidos à cirurgia de revascularização do miocárdio (CRM), comparando aqueles que realizaram o procedimento àqueles sem CEC.

Durante um período de 15 meses, os pesquisadores avaliaram 22 pacientes com mais de 18 anos de idade, excluindo aqueles com doença renal crônica preexistente, doença valvular, cirurgia cardíaca prévia ou transplante, ou exposição recente a agentes nefrotóxicos. A quantificação dos biomarcadores foi realizada por meio de técnicas ELISA. Os autores mensuraram nefrina, KIM-1, MCP-1 e NGAL urinários e syndecan-1 (um marcador de degradação do glicocálice endotelial) e NGAL séricos.

Do total da coorte, 9 pacientes (41%) foram submetidos à CEC. Embora o tamanho da amostra seja limitado e somente análises univariadas tenham sido realizadas, o estudo fornece uma visão crítica: os níveis basais de biomarcadores (antes da cirurgia) foram semelhantes entre os grupos; porém, após o procedimento cirúrgico, houve um aumento significativo da nefrina urinária e da NGAL (urinária e sérica) nos pacientes submetidos à CEC. Esses achados sustentam a hipótese de que a CEC pode desempenhar um papel direto no desencadeamento da injúria renal e endotelial, contribuindo para a natureza multifatorial da IRA nesse contexto.

Entretanto, algumas limitações metodológicas devem ser mencionadas. O grupo submetido à CEC apresentou escores STS (*The Society of Thoracic Surgeons Operative Risk Calculator*) mais elevados, disfunção ventricular esquerda mais acentuada e, provavelmente, foi composto por pacientes com anatomia coronariana mais complexa (por exemplo, doença de múltiplos vasos, aterosclerose difusa, artérias de pequeno calibre ou de difícil acesso). Essas características aumentam inerentemente o risco do procedimento e podem atuar como fatores de confusão. Além disso, não foi claramente explicado como foi tomada a decisão de designar os pacientes para CEC ou CRM sem CEC, levantando a possibilidade de viés de seleção. Uma vez que a escolha da estratégia cirúrgica provavelmente reflete a complexidade do caso e o estado cardiovascular subjacente, essa alocação não randomizada pode ter afetado os desfechos observados, particularmente no que diz respeito à elevação dos biomarcadores e ao risco de IRA.

Além disso, o uso da equação CKD-EPI no contexto agudo, embora comumente aplicado, não é totalmente validado para a detecção precoce de alterações agudas na função renal.

Apesar dessas limitações, o principal ponto forte do estudo reside em sua capacidade de demonstrar a liberação diferencial de biomarcadores somente após o início da CEC, reforçando o possível papel da CEC na contribuição para a injúria renal. Os dados apresentados oferecem uma contribuição valiosa para o crescente campo da avaliação de risco renal baseada em biomarcadores na medicina perioperatória.

## Rumo à Medicina de Precisão na IRA: Estratificação e Prevenção

É importante que a nefrologia avance em direção à medicina de precisão, especialmente em cenários clínicos que envolvem múltiplos estímulos lesivos simultâneos, como ocorre em cirurgias cardíacas. A identificação do insulto predominante em um determinado paciente pode viabilizar intervenções direcionadas, mitigar a injúria renal de forma mais eficaz e, por fim, melhorar os desfechos. Nesse contexto, os biomarcadores não são apenas ferramentas para detecção precoce, mas também para discriminação fisiopatológica, estratificação de risco e planejamento terapêutico personalizado.

Estudos futuros com amostras maiores e ajuste multivariado para variáveis de confusão são essenciais. Além disso, a inclusão de desfechos como a recuperação renal em longo prazo ou a necessidade de terapia renal substitutiva fortaleceria a aplicabilidade clínica desses achados.

Como nefrologistas, devemos defender estratégias multidisciplinares que integrem a vigilância por biomarcadores, a otimização hemodinâmica e os protocolos de preservação dos rins em pacientes cirúrgicos de alto risco. A predição precoce deve se tornar a pedra angular no manejo da IRA, transformando nossa abordagem de reativa para preventiva.

## Data Availability

Nenhum dado foi gerado ou analisado para esse editorial.
